# Dynamical diagnostic of extreme events in Venice lagoon and their mitigation with the MoSE

**DOI:** 10.1038/s41598-023-36816-8

**Published:** 2023-06-28

**Authors:** Tommaso Alberti, Marco Anzidei, Davide Faranda, Antonio Vecchio, Marco Favaro, Alvise Papa

**Affiliations:** 1 Istituto Nazionale di Geofisica e Vulcanologia, via di Vigna Murata 605, 00143 Rome, Italy; 2 Marine Archaeology Research, 30100 Venice, Italy; 3 Laboratoire des Sciences du Climat et de l’Environnement, CEA Saclay l’Orme des Merisiers, UMR 8212 CEA-CNRS-UVSQ, Université Paris-Saclay & IPSL, 91191 Gif-sur-Yvette, France; 4 London Mathematical Laboratory, 8 Margravine Gardens, London, W6 8RH UK; 5 LMD/IPSL, Ecole Normale Superieure, PSL Research University, 75005 Paris, France; 6 Radboud Radio Lab, Department of Astrophysics/IMAPP-Radboud University, Nijmegen, The Netherlands; 7 LESIA, Observatoire de Paris, Universite PSL, CNRS, Sorbonne Universite, Universite de Paris, 5 Place Jules Janssen, 92195 Meudon, France; 8 Centro Previsioni e Segnalazioni Maree, 30100 Venice, Italy

**Keywords:** Climate-change impacts, Climate-change mitigation, Statistical physics, thermodynamics and nonlinear dynamics

## Abstract

Extreme events are becoming more frequent due to anthropogenic climate change, posing serious concerns on societal and economic impacts and asking for mitigating strategies, as for Venice. Here we proposed a dynamical diagnostic of Extreme Sea Level (ESL) events in the Venice lagoon by using two indicators based on combining extreme value theory and dynamical systems: the instantaneous dimension and the inverse persistence. We show that the latter allows us to localize ESL events with respect to sea level fluctuations around the astronomical tide, while the former informs us on the role of active processes across the lagoon and specifically on the constructive interference of atmospheric contributions with the astronomical tide. We further examined the capability of the MoSE (Experimental Electromechanical Module), a safeguarding system recently put into operation, in mitigating extreme flooding events in relation with the values of the two dynamical indicators. We show that the MoSE acts on the inverse persistence in reducing/controlling the amplitude of sea level fluctuation and provide a valuable support for mitigating ESL events if operating, in a full operational mode, at least several hours before the occurrence an event.

Anthropogenic climate change is seriously impacting our society in terms of natural disasters and economic damages, claiming for urgent mitigating strategies^1^. One of the most affected areas are surely coastal zones which are particularly exposed to extreme sea levels, likely projected to increase in the future with increasing magnitude, duration, and frequency^2^. This is the case of the historical city of Venice and its lagoon in the Northern Mediterranean Sea which are continuously exposed to flooding events, thus requiring for a realistic assessment of risks and their future projections^3^. Indeed, Extreme Sea Level (ESL) events in Venice produced significant damage to the cultural and economic heritage of the city over the years, involving the flooding of the city’s streets and buildings, and also causing widespread damage to artworks, including the historic St. Mark’s Basilica, or damage to the city’s cultural landmarks and historic shops^4^. The main causes of ESLs and flooding in Venice must be searched in the constructive interference of different factors as the astronomical tides, seiches, and atmospheric forcings (e.g., strong winds and depressions, storm surges, meteotsunamis) that, individually and/or collectively acting, can increase the probability of occurrence of extreme events^3^. Typically, the highest sea levels and storm surges occur in close correspondence with southeasterly wind (sirocco), occurring from November to March. These surges can be related to extratropical cyclones in the Alpine region, although the most intense events are associated with phenomena located in the western Mediterranean (around the Gulf of Genoa), while a small fraction is driven by atmospheric circulation patterns of related to the variability over the Euro-Atlantic sector^3^. Although the main causes of extreme sea levels are well understood, the intrinsic complexity of the climate system and the current facing with climate change makes impossible to provide reliable forecasts, claiming for novel efforts in evaluating and anticipating incipient transitions in the lagoon dynamics by developing new strategies to support and inform on extreme sea levels^5,6^.

Climate change is indeed one of the main drivers of extreme flooding in Venice that could become more dangerous due to the projected rising sea levels in Venice expected by the end of the 21th century. Indeed, sea levels in Venice have risen by around 26 centimeters over the past century, with projections indicating that they could rise by an additional 50 centimeters at 2100^3^ and according to Vecchio et al.^7^ up to $82 \pm 25$ cm. To address these challenges, the Italian government has invested about 6.2 billions of euros in the Experimental Electromechanical Module (MoSE) project. It consists of a series of mobile barriers that can be raised up to temporarily isolate the Venice Lagoon from the Adriatic Sea and designed to protect the city from flooding^8–10^. These barriers are located at the three inlets that connect the lagoon with the north Adriatic Sea (Chioggia, Malamocco and Lido). Although the project has faced some delays and controversies, the barriers were successfully tested in 2020 and are expected to become fully operational in the near future (https://www.mosevenezia.eu/?lang=en). The barriers are normally submerged in the water and are raised in anticipation of flood events (where sea levels within the lagoon are expected to be over than 110 cm). Since its operative phase (end of 2020), barriers’ closures have often lasted between a few hours and 1 day, with an average closing time of about 4/5 h, and have been activated more than 50 times (https://www.mosevenezia.eu/?lang=en). However, since the issue of Acqua Alta in Venice is a complex and multifaceted problem that requires a coordinated and sustained effort to address, the MoSE project represents an important step forward. Despite the high cost of maintenance (about 63 million of euros per year) it is clear that further action is needed to mitigate the impacts of climate change and protect this unique and valuable cultural heritage site. This requires coordinated action from multiple stakeholders, including policymakers, scientists, and local communities that, by working together, can actively contribute to reduce the risks posed by extreme events and ensure the long-term sustainability of this unique and valuable city and its lagoon.

The aim of this contribution is to propose a novel approach in diagnosing extreme events in Venice lagoon and in also proposing a way to inform on the role of the MoSE in a dynamical approach by using recent developments of extreme value theory (EVT). Indeed, while EVT provides a suitable theoretical background to estimate the probability of returns of extreme events (i.e., events that are large or small relative to some specific threshold), widely used in several contexts^11^ including ESLs in Venice^12^, it is not suitable to deal with persistent or rare phenomena as spatially extended patterns triggering extreme events. This has motivated the introduction of a new mathematical formalism based on defining extreme events as rare recurrences in the state-space of a high-dimensional system^13^ bridging together the statistics (i.e., the traditional extreme value theory) and the dynamics (dynamical systems theory) of extreme events. One of the main achievements of this combined approach is to characterize rare/extreme events by means of two metrics able to inform on the predictability (instantaneous dimension^14^), persistence and synchronization (inverse persistence^15^) of physical states.

In this study we use tide gauge data at hourly resolution from 4 stations across the Venice lagoon (Chioggia Cittá-Vigo, Malamocco Porto, Punta della Salute Giudecca, and Laguna Nord Saline) during the period from 01-Jan-2005 to 01-Jan-2022 (see Section Methods). The station distribution that spatially cover the entire lagoon and the availability of long time series of sea level data, allow us to have a sufficiently large statistics ($\sim 1.5 \times 10^5$ data points) and to compare the dynamics of several ESL events with and without the MoSE. Figure 1 reports the seal levels (SL) measured at the 4 selected stations.

**Figure 1:**
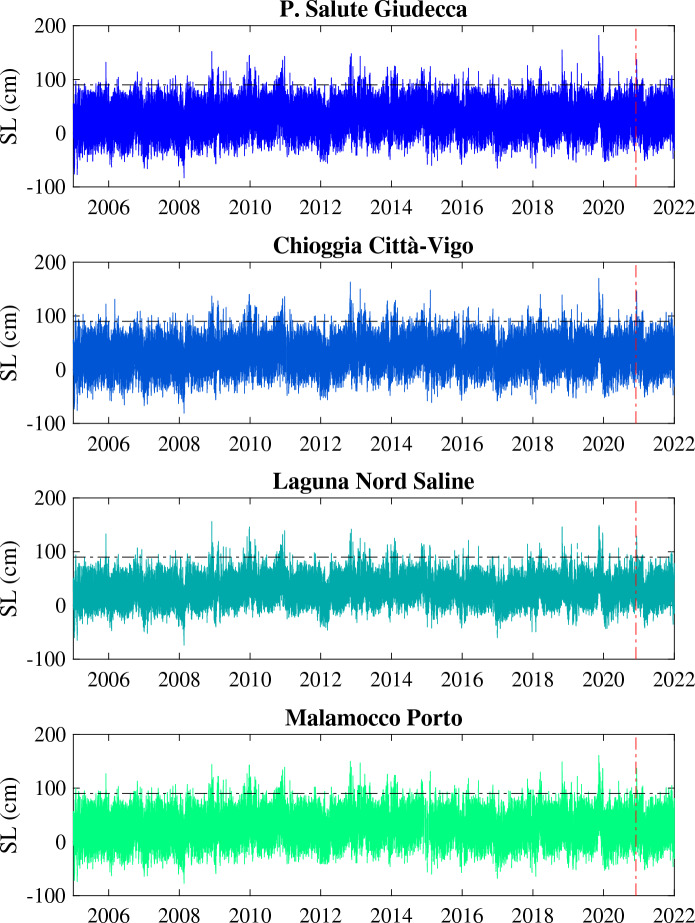
The sea level (SL, in centimeters) measured at the 4 selected stations across the Venice lagoon (Chioggia Cittá-Vigo, Malamocco Porto, Punta della Salute Giudecca, and Laguna Nord Saline). The horizontal black dashed-dotted line marks the threshold of the 99th percentile of sea levels used to select extreme events and the vertical dashed-dotted red line indicates the entry into operations of the MoSE.

Several ESLs have been recorded with small differences from one station to another, depending on their location and on the response of the lagoon itself. During the investigated period, the tide gauge located at Punta della Salute recorded 72 ESLs with SL between 110 and 120 cm, 26 ESLs between 120 and 130 cm, 9 ESLs between 130 and 140 cm, and 14 ESLs with SL$\ge 140$ cm (see, e.g., https://www.comune.venezia.it/it/content/grafici-e-statistiche). An exceptional ESL event occurred on 12 November 2019 with the highest sea level value of 187 cm recorded at Punta della Salute, representing the second highest level since 1872. This extreme event was characterized by the superimposition of four phenomena: (i) the peak of the astronomical tide, (ii) the persistence of a high mean sea level of the Adriatic due to a strong low-pressure system, (iii) a strong south-easterly wind (sirocco), and (iv) the passage above the Venice lagoon of a small cyclone with local winds over 100 km/h^16^. Furthermore, in the same week of November 2019 the sea level exceeded 140 cm 4 times, while in the whole 2019, the sea level exceeded 110 cm at least 28 times, with a total permanence of around 50 h in the month of November, causing the 12% of the city flooded.

By using together the 4 stations, as to form a 4-D state-space, we evaluate the instantaneous dimension *d* and the inverse persistence $\theta$ during the investigated period to retrieve information on the active number of degrees of freedom and the repeatability and the persistence of the different states of the system (see Section Methods for details). Figure 2 shows the scatter plot of *d* versus $\theta$ as a function of the sea level measured within the lagoon at the reference station of Punta della Salute.

**Figure 2:**
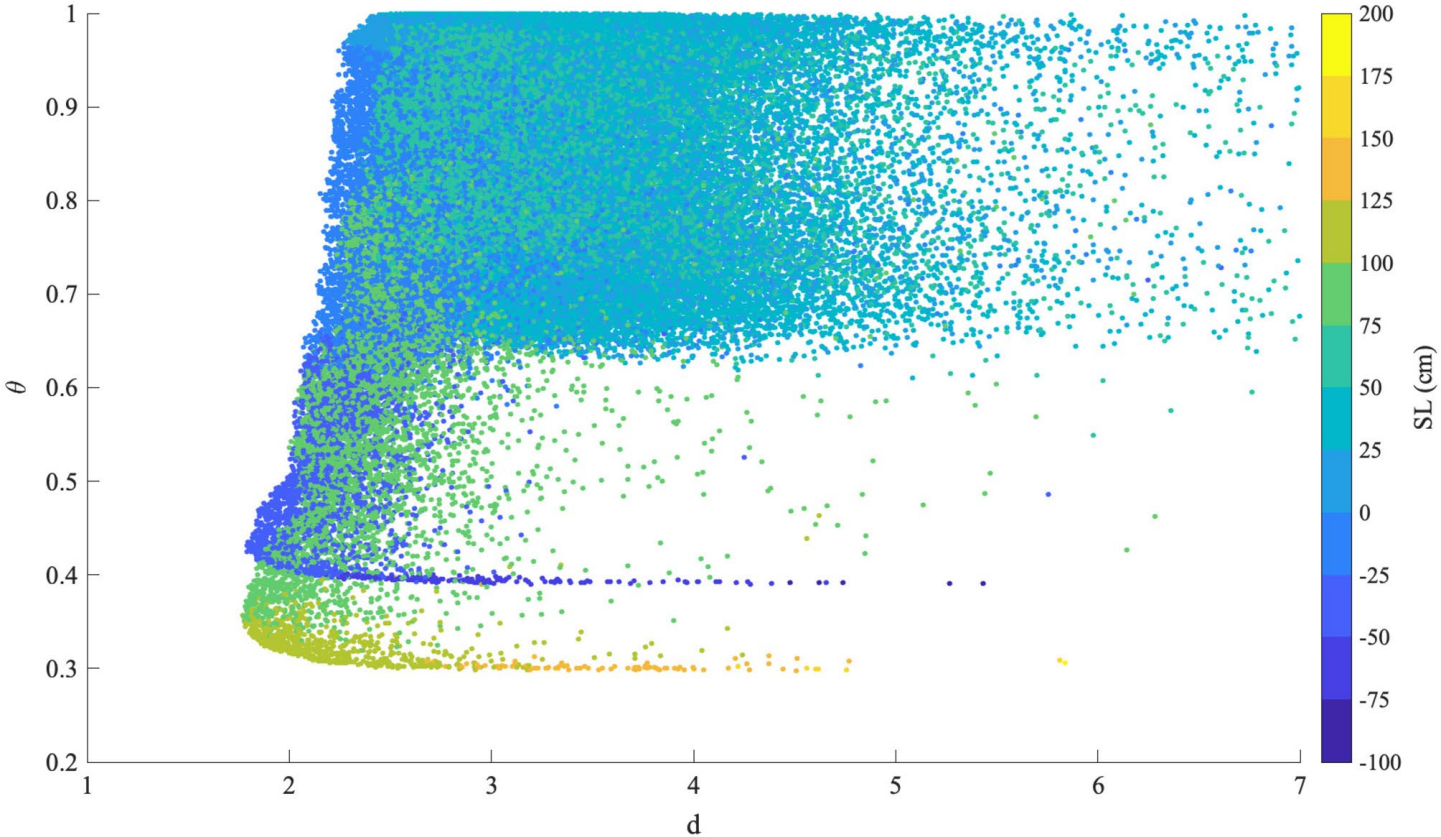
The scatter plot the dimension-persistence *d*-$\theta$ of the hourly values of the instantaneous dimension *d* and the inverse persistence $\theta$. The color-bar refers to the sea level measured at Punta della Salute.

The greater fraction of states *s* is characterized by high values of $\theta$ ($>0.6$) and a range of dimensions *d* within 2 and 5, corresponding to sea levels ranging between $-0.25$ and 0.75 m. These dynamical states *s* are related to fluctuations around the semi-diurnal astronomical tide: the neap tide contribution (first and last quarter), with a smaller range of variability, and the spring tide (new/full moons) where larger values of the sea levels are typically reached (spring tides). These states are characterized by a low persistence since they are representative of fluctuations around the quasi-periodic dynamics induced by the astronomical tide. The most interesting behavior is however observed for the most extreme events, i.e., those reaching larger values of sea levels, as shown by the dark and light yellow spots in Fig. 2. They are localized in a thin band in the $d-\theta$ plane with an almost constant value $\theta \simeq 0.3$ and an increasing *d* as the SL increases. These events are then associated with the most persistent conditions and are representative of the ESL events observed in the Venice lagoon. Furthermore, a similar trend is observed for the most negative (lowest) sea levels, with increasing dimension as the sea level decrease towards extreme negative values but with a slightly larger value of the inverse persistence, $\theta \simeq 0.4$.

To further exploit these two different bands, since they are characterized by larger (in terms of absolute) values of sea levels, we selected the ESLs recorded outside the lagoon since 2005 with sea level greater than 110 cm. The list of extreme events is freely available at https://www.comune.venezia.it/it/content/grafici-e-statistiche. Figure 3 reports the location of these ESL events in the *d*-$\theta$ plane.

**Figure 3:**
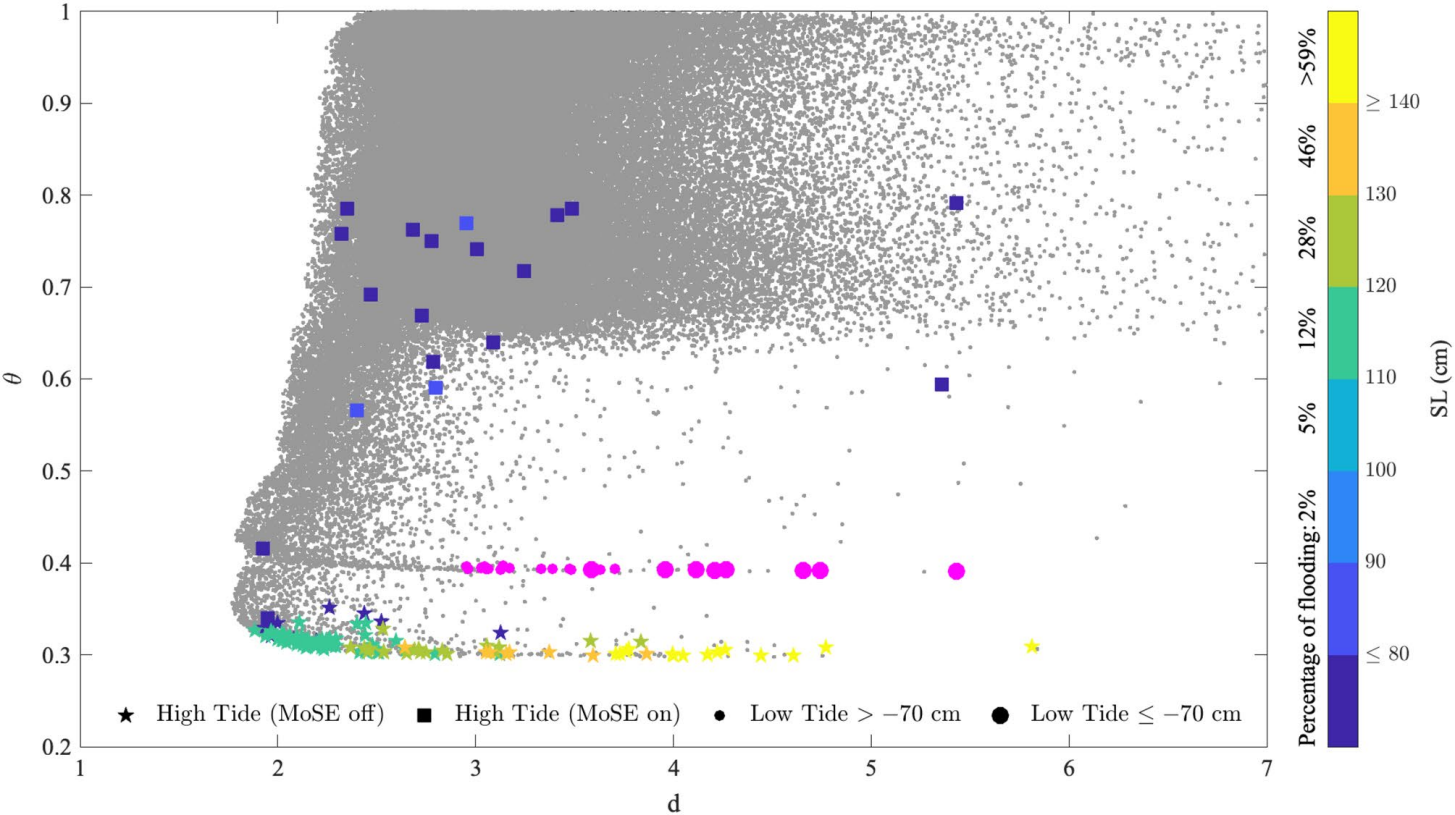
The scatter plot *d*-$\theta$ of the hourly values (gray dots) of the instantaneous dimension *d* and the inverse persistence $\theta$. Stars indicate positive ESL events with no MoSE activation, squares refer to positive ESL events when the MoSE was operating, filled magenta circles indicate negative ESL events. The larger is the size of filled circles the more negative is the corresponding sea level. The colored stars and squares correspond to hourly values $(d, \theta )$ for each identified positive ESL event where colors refer to the sea level measured at Punta della Salute corresponding to different percentage of flooding.

We clearly observe that positive ESL events (SL$\ge 110$ cm, stars in Fig. 3), corresponding to percentages of flooding larger than 12%, are all localized in the thin band with $\theta = 0.3$, with a clear increasing dimension *d* as the increase of flooding area/percentage. A similar behavior, with increasing dimensions as decreasing towards negative ESLs is observed, although all located in a different band with $\theta = 0.4$. This suggests that the inverse persistence $\theta$ can be helpful to discriminate between ESLs and sea level fluctuations around the astronomical tide. Indeed, the former are located in a specific thin band with low $\theta$ values, while the latter spread out over a wider range of $\theta$ values, mainly with $\theta > 0.6$. By further looking at the ESL events the increasing of the dimension indicates additional degrees of freedom in the system which can be related to external forcings to the system. These forcings are clearly related to changes into the atmospheric weather regimes, related to depressions in the synoptic atmospheric pressure and strong winds leading to storm surge, meteotsunami, PAW surge, and seiches^3^. Thus, the instantaneous dimension *d* can inform us on the role of active processes across the lagoon and specifically on the constructive interference of atmospheric contributions with the astronomical tide. Indeed, we note that the largest dimension ($d = 5.81$) corresponds to the 12 November 2019 event produced by the combined effect of a meteotsunami and a local surge within the lagoon both in phase with the astronomical tide^4,16^ . Instantaneous dimensions $d>4$ are associated with the ESLs reaching SL$\ge 140$ cm with at least two different surge contributions coming from storm surge and seiches or storm and surge caused by long planetary atmospheric waves (PAW), contributing (at least one of the two) in a constructive way with the astronomical tide. ESL events associated with dimensions $d \in [2,4]$ result instead from storm surge or low-frequency signals or high seiches of the Adriatic Sea with in-phase relations with the astronomical tide. Our results are well in agreement with the statistical analysis proposed by Ferrarin et al.^4^ showing that the dominant contribution is attributed to storm surge, while PAW surge and inter-decadal, inter-annual and seasonal sea level variability (IDAS) typically produce less severe events. Our findings also support recent results obtained in the framework of atmospheric circulation, showing that larger values of the dimensions are usually related to a blocking dynamics of the atmosphere^17^ which is the main responsible of ESL events^3^.

All these ESL events share a common feature: the MoSE was not operating. When, instead, the MoSE was active, with at least one barrier raised, a completely different behavior is observed in terms of the pair $(d, \theta )$, as reported by filled squares in Fig. 3. Lower sea levels ($< 90$ cm) are detected within the lagoon, despite the higher levels registered outside (larger than 110 cm): this means that the MoSE effectively reduces sea levels within the lagoon (as expected). However, there is a wide spread in the *d*-$\theta$ plane, without a clear indication on a specific pattern characterising events where the MoSE was operating. Since the MoSE directly acts on the degrees of freedom of the lagoon by reducing/cancelling out the dynamics of the North Adriatic it should be expected that the instantaneous dimension *d* reduces and, at the same time, also the inverse persistence $\theta$ should reduce. This is not the case, apart two events with $d \simeq 2$ and $\theta = \{0.34, 0.32\}$, respectively. The two cases corresponds to 06 December 2020 and 09 February 2021 during which the MoSE system was operating at least 24 h before the event itself, thus completely separating the lagoon from the external sea circulation, with the lagoon forming a closed basin, independent on the external conditions. Indeed, during these two cases the contribution coming from the astronomical tide is practically absent. The great majority of MoSE events is associated with inverse persistence $\theta$ between 0.6 and 0.8 and dimension $d \in [2,4]$. This suggests that the MoSE is acting on the inverse persistence $\theta$ instead on the active number of degrees of freedom *d*, which is consistent with our results on the role of $\theta$ in discriminating ESLs. This means that the MoSE is able to reduce/control the amplitude of sea level fluctuations superimposed to the astronomical tide, instead of directly acting on the degrees of freedom (although reduced with respect to those observed during ESL events where $d>4$). Finally, there are two cases, corresponding to 30 December 2020 and 06 November 2021, which are characterized by larger values of both $\theta$ and *d* that cannot be attribute to a specific class, i.e., they are not ESL events since $\theta > 0.6$ nor sea level fluctuations around the astronomical tide ($d>4$). These two events were characterized by the MoSE only partially operating (with only one barrier raised up), still allowing a non-negligible contribution coming from the Adriatic sea. Thus, the two metrics $\theta$ and *d* are also particularly helpful for detecting the role of the MoSE in controlling the sea level within the lagoon.

To further inspect the role of the instantaneous dimension *d* in disentangling extreme events of different origin we selected two specific time intervals corresponding to two major storms that hit Venice on 29 October 2018 and on 12 November 2019. Figure 4 reports the behavior of the instantaneous dimension *d* along the trace observed by the tide gauge of Punta della Salute during the three selected events.

**Figure 4:**
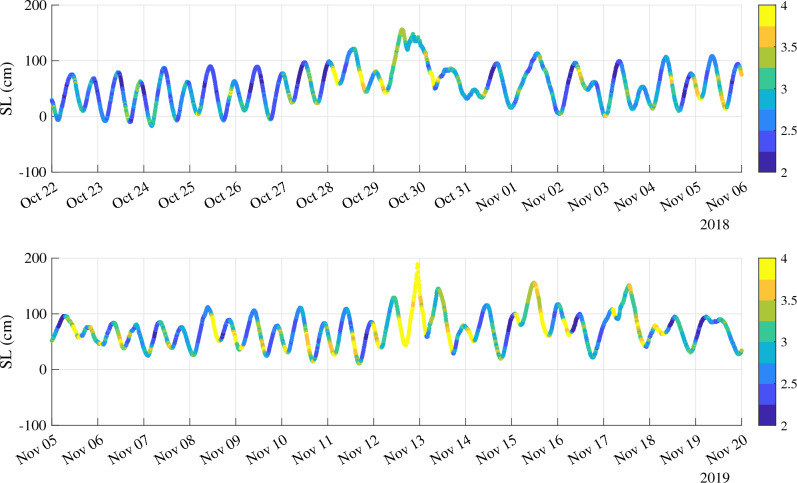
The behavior of the instantaneous dimension *d* (colorbar) along the trace observed by the tide gauge of Punta della Salute during the two selected events: the 29 October 2018 (upper panel) and the 12 November 2019 (lower panel). The color-bar is saturated to 5 for visual purposes.

It is clear that a larger dimension *d* is observed for the 2019 event with respect to the 2018 one that can be linked with the different morphology of both events. Indeed, the 2018 storm was the results of strong southeastern sirocco wind along the Adriatic Sea following a low pressure system in the western Mediterranean^18^. This resulted in a storm surge more than 140 cm peaking during the trough of the astronomical tide and in a maximum observed sea level of 154 cm. This ESL was enough to flood 70% of the town but if the storm hit a few hours earlier, Venice would have experienced by far the worst flood in its history ($> 210$ cm). It is interesting to note that indeed before the peak the dimension is larger than the peak that could be attributed to the different synchronizations (interference) of the components contributing to the sea level. This seems to suggest that the dimension is sensitive to external perturbations (especially storm surge) and it can be used to dynamically diagnose the role of external forcings and their phase-relation (synchronization) with the astronomical tide contribution.

Conversely, the 2019 event resulted from a combination of factors, each unexceptional when viewed separately, except for the wind during the evening. The storm surge following the sirocco wind was “only” 70 cm, then with a smaller sea level when compared to many other historical events. However, the southeasterly winds coincided with one of the full moon astronomical tidal peaks. All of this was superimposed on a higher than usual local sea level due to a prolonged low atmospheric pressure anomaly in the Mediterranean, in particular over the Adriatic Sea. Finally, the strong winds driven by the fast-moving depression pushed the lagoon’s water against the south face of Venice. Thus, the constructive interference of the different components produced a maximum observed level of 189 cm and flooding of 85% of the town and, in terms of the dimension, it produced the largest value of *d*, being as described above *d* a measure of the constructive interference of the different contributions.

In this work we reported, for the first time, a novel approach for detecting, diagnosing, and classifying Extreme Sea Levels in the Venice lagoon by means of two dynamical indicators based on combining extreme value theory and dynamical systems. This method is particularly suitable for extreme events that are triggered by spatially extended patterns as the response of the Venice lagoon to atmospheric patterns related to depressions and strong winds. Indeed, our two metrics are able to instantaneously capture and characterize the different features of the system, instead of providing a probability of return of a specific event above a selected threshold^11,12^. Thus, they provide a time-dependent picture of the system, thus yielding a dynamical diagnostic of the lagoon conditions based on the value of the instantaneous dimensions *d* and the inverse persistence $\theta$. We found a close connection between the occurrence of ESLs and the inverse persistence, while the instantaneous dimension informs us on the number of interfering factors contributing to the increase of sea level. Specifically, when the Venice historical city centre is flooded at least for its 12%, corresponding to sea levels greater than 110 cm, the value of the inverse persistence $\theta$ localize in a thin band with constant value $\theta \simeq 0.3$, with an increasing dimension *d* as the flooding area/percentage increases. The increasing of the dimension, suggesting additional degrees of freedom, reflects the role of external forcings to the system induced by specific spatially-extended atmospheric blocking regimes and patterns (depressions in the synoptic pressure and strong winds) leading to storm surge, meteotsunami, PAW surge, and seiches^3^. Thus, we show that the inverse persistence $\theta$ allows us to localize ESL events with respect to sea level fluctuations around the astronomical tide, while the instantaneous dimension *d* informs us on the role of active processes across the lagoon and specifically on the constructive interference of atmospheric contributions with the astronomical tide. Our findings also support recent results obtained in the framework of atmospheric circulation, showing that larger values of the dimensions are usually related to a blocking dynamics of the atmosphere which is the main responsible of extreme events^17^. Finally, by inspecting the values of the two dynamical indicators during ESL events mitigated via the MoSE system we provide also a quantitative measure of the role of this safeguarding system. We found that when the MoSE is operating at least 24 h before the event itself a significant reduction of the degrees of freedom ($d \simeq 2$) and an increase in the stability of the lagoon ($\theta \simeq 0.32$) is observed. This indicates that the MoSE, separating the lagoon dynamics from the North Adriatic one, is able to generate a closed basin which is independent on external conditions, thus safely protecting the historical centre and effectively stabilizing the level of the tide, also reducing/cancelling out the astronomical tide. However, this is only valid for 2 cases over 19 ($\simeq 10\%$), while the majority of events (15/19) are characterized by higher values of the inverse persistence ($\theta \simeq 0.7$). This indicates that the MoSE acts on the inverse persistence $\theta$ instead on the active number of degrees of freedom *d*, which is consistent with our results on the role of $\theta$ in discriminating ESLs from fluctuations around the astronomical tide, by reducing/controlling the amplitude of sea level fluctuations. Finally, there are other two cases where the instantaneous dimension exceeds the values observed for ESL events ($d>5$) and with large $\theta$ values ($> 0.6$) which corresponds to a partially-operating MoSE (with only one barrier raised up), still allowing a non-negligible contribution coming from the Adriatic Sea, thus affecting the stability and the number of degrees of freedom within the lagoon.

The present study shows only a first attempt to demonstrate the potential of dynamical system metrics based on extreme value theory in diagnosing and classifying extreme events and their possible source mechanisms and triggering processes. Our results support the idea to use these metrics for a real-time monitoring of the dynamics of the lagoon when they are only based on tide gauge data as well as by looking at the full dynamical component, without discerning the different contributions coming from different sources as the astronomical tide, storm surge, seiches, etc. The main novelty introduced here is the possibility of using $\theta$ to discern ESL events and *d* to characterize the constructive interference between external forcing components and the astronomical tide, thus informing on the sea level peaks reached during ESL events. The two metrics provide an instantaneous view of the system that can represent a valuable support for nowcasting and real-time monitoring of sea levels within the lagoon, informing us on the contribution of atmospheric factors and their synchronization with the astronomical tide. Indeed, they can provide a real-time picture of the occurrence of ESLs by looking at the specific trajectory they follow in the $d-\theta$ plane. Based on Fig. 2 indeed we observe that typically the portion of the $d-\theta$ plane occupied during “normal” activity is the $d \in [2,4]$ and $\theta >0.6$, migrating firstly towards lower $\theta$ and lower *d* and then with increasing *d* as the sea level increases with constant $\theta$. Thus, by inspecting in a real-time way the behavior in the $d-\theta$ plane our metrics can support nowcasting operations. However, in order to provide a support for forecasting the lagoon dynamics and for flood risk management it would be desirable to investigate the dynamics of some atmospheric/meteorological variables (as the sea level pressure or wind speed and direction) to both provide attribution of the observed events to the climate change as well as to inform on the predictability of ESLs, supporting weather forecasting system in an operation manner^19–21^ and for developing mitigation strategies^22^. Further studies are also needed to investigate the multi-scale variability of sea levels and how the different scale-dependent components contribute to the observed values of both metrics. This can be achieved by looking at refined analysis tools for detecting state- and scale-dependent properties of physical systems that are vastly encountered in climate sciences^17,23–25^. Finally, a crucial point is also to investigate the expected future scenarios due to the combined effects of sea level rise^7^ and increasing number of extreme weather phenomena^1^ and how our metrics can support operative protocols for safeguarding the lagoon via the MoSE system in a changing future climate.

## Methods

### Instantaneous dimension

A dynamical systems approach is applied to investigate the persistence and the predictability of extreme events in the Venice lagoon. The idea is that the 4-D vector $x(t) = \{x_i(t)\}_{i \in [1,4]}$, made by the instantaneous values of the 4 selected stations, is representative of the states of the system, i.e., of the lagoon, at the time *t*. The main goal of the analysis is to characterize the statistics of a given system’s state *s* in terms of its repeatability/recurrence (i.e., how many times the system is found in this state) and its residence/stability (i.e., for how long the system stays in this state) by exploiting the link between the extreme value theory and the Poincaré recurrence theorem^13^. This can be done by considering the state *s* and all other possible states *x*(*t*) whose Euclidean distance $\delta (t)$ is below a certain threshold $\varepsilon$, i.e.,

1 $$\begin{aligned} \delta (t) \doteq \left| s - x(t)\right| < \varepsilon . \end{aligned}$$

The instantaneous properties of the state *s* can be described by two metrics: the instantaneous dimension *d*(*s*) and the inverse persistence $\theta (s)$. They are derived by fitting the probability of returning in a hyper-sphere of radius $\varepsilon$ centered on *s* as a Generalized Pareto-like Distribution (GPD) as proven by the Freitas-Freitas-Todd theorem^26^ properly adapted by Lucarini et al.^14^. This means that by defining $g(t) = -\log \left( \delta (t) \right)$ then the probability of logarithmic returns is

2 $$\begin{aligned} \mathscr {P}\left( g(t) > q, s\right) \simeq \exp {\left[ -\frac{x(t) - \mu (s)}{\sigma (s)} \right] } \end{aligned}$$

whose parameters $\mu (s)$ and $\sigma (s)$ depends on the selected state *s* and are respectively related to the 1st and the 2nd moments of $\mathscr {P}$, while the statistical threshold *q* is related to the Euclidean threshold $\varepsilon$ via the relation $q = \exp (-\varepsilon )$. This means that the condition (1) is equivalent to requiring that *g*(*t*) is over the threshold *q* that can be set as a percentile of *x*(*t*)^13,27^. The instantaneous dimension is simply obtained as the inverse of $\sigma (s)$, i.e.,

3 $$\begin{aligned} d(s) = \sigma (s)^{-1}, \end{aligned}$$

and, when all available states are explored (i.e., all time instants are considered), an instantaneous view of the repeatability of the system’s states is obtained. In a similar fashion to the usual concept of dimension introduced in the framework of dynamical systems^28–30^, the distribution of instantaneous dimensions provides us powerful information on the predictability of observed states, helping us in characterizing the overall instantaneous dynamics of the system^13,17,31^.

### Inverse persistence

The inverse persistence $\theta (s)$, providing us information on the residence time of the system in a given state *s*, can be instead evaluated by using the Süveges maximum likelihood estimator for the extremal index^15,32^

4 $$\begin{aligned} \theta = \frac{\sum _{i=1}^N \rho S_i + N - 1 + N_c - \left[ \left( \sum _{i=1}^{N-1} \rho S_i + N - 1 + N_c\right) ^2 - 8 N_c \sum _{i=1}^{N-1} \rho S_i \right] ^{1/2}}{2 \sum _{i=1}^{N-1} \rho S_i} \end{aligned}$$

with *N* being the number of observations exceeding a defined threshold, $\rho$ is the distribution function of the selected threshold, $S_i$ is the exceedance distance, and $N_c = \sum _{i=1}^{N-1} I(S_i\ne 0)$ where *I* is the indicator function for the selected $S_i$. The reader is referred to^32^ for further details on the calculation. The extremal index $\theta$ tells us on the finite amount of time spent by the system in the vicinity of the state *s*, i.e., it is a measure of the inverse of the mean residence time within the hyper-sphere of radius $\varepsilon$, such that it can be introduced in Eq. (2) as

5 $$\begin{aligned} \mathscr {P}\left( g(t) > q, s\right) \simeq \exp {\left[ -\theta (s) \; \frac{x(t) - \mu (s)}{\sigma (s)} \right] }. \end{aligned}$$

Since $\theta (s) \in [0,1]$, as much as $\theta (s) \rightarrow 0$ the more persistent is the state *s*; conversely, when $\theta (s) \rightarrow 1$ the state *s* is unstable and the system immediately leaves *s*^13,15^. As for the instantaneous dimension by exploring all states (i.e., all time instants), an instantaneous view of the persistence of the system into the different states is obtained. Thus, each state *s* of the system, corresponding to the time instant *t* of the time series, is now described by the pair $(d, \theta )$. These metrics have provided novel insights and a different view of several geophysical extreme phenomena as transient events in the atmospheric circulation^33–36^, in the ocean dynamics^27,37^, transient disturbances of the geomagnetic field due to geomagnetic storms and magnetospheric substorms^38^, and earthquake dynamics^39^. In the following we select the $q = 99$th percentile of sea levels corresponding to at least 2% of flooding (i.e., $\ge 90$ cm). This value is in agreement with previous studies^4^. Furthermore, since Venice lagoon experienced a significant relative sea level raise, both in the last century and accelerating during the last 20 years, to assess the robustness of our results we also carried out the analysis on the detrended time series of the tidal level. The detrending has been performed by using the Empirical Mode Decomposition (EMD)^40^, an adaptive decomposition method allowing us to preserve the non-stationary and non-linear properties of time series by extracting a monotonic nonlinear trend. The reader is referred to previous papers for more details^41^. A positive trend has been filtered out, whose range of variability ($+7$ cm for Punta Salute, $+8$ cm for Chioggia Cittá-Vigo, $+3$ cm for Laguna Nord Saline, and $+2$ cm for Malamocco Porto) is well in agreement with estimations provided by Ferrarin et al.^4^ using a 19-year running mean. The extrapolated values for *d* and $\theta$ are statistically comparable ($p-$value>0.05) under the null-hypothesis based on the non-parametric Kolmogorov-Smirnov test both for the raw and the detrended time series. Finally, since our analysis is focused on 17 years the results are completely free from any other long-term contribution due to multi-decadal variability and non-linear tidal effects due to the lunar cycle^3^.

### Tide gauge data

**Figure 5:**
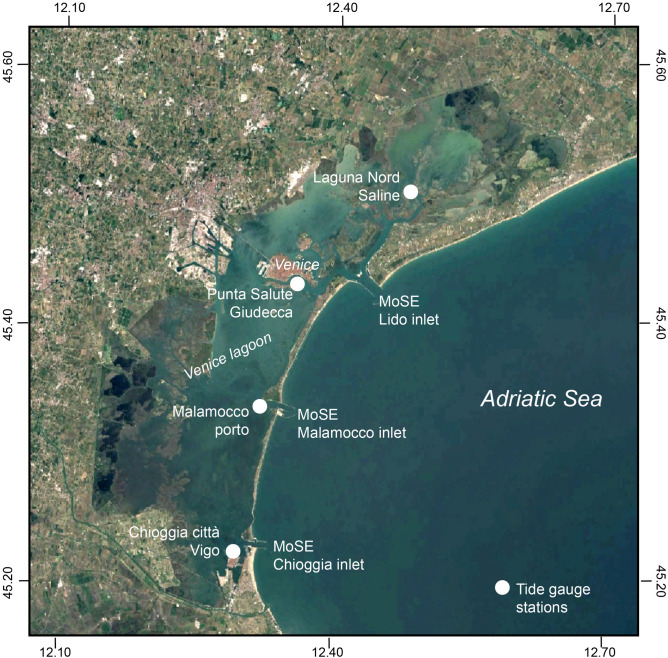
The geographical location of the 4 selected tide gauge stations (filled white circles) across the Venice lagoon (Chioggia Cittá-Vigo, Malamocco Porto, Punta della Salute Giudecca, and Laguna Nord Saline) together with the three MoSE inlets. The map has been produced with Google Earth.

We use tide level data from the Tidal gauge network of the Tidal forecast and Information Center, which consists of 16 automatic stations located in the Venice lagoon and on the Venetian coast. The tide level data at hourly resolution are freely available at https://www.comune.venezia.it/it/content/dati-dalle-stazioni-rilevamento. Four stations (Punta Salute, Lido Diga Sud, Malamocco Diga Nord, and Chioggia Diga Sud) are collecting data at least since 1984, while other stations are collecting data only during the last 10–20 years. The geographical location of the four selected stations is reported in Fig. 5. Tidal heights, reported in meters, refer to the tidal zero of Punta Salute. In this work we are interested in investigating the dynamics within the lagoon to determine its dynamical properties and how they can be altered by the use of the MoSE. To avoid spurious redundancy of information due to stations too close together, to minimize the number of data gaps ($<1.5 \%$) and to maximize information on the spatial variability of the lagoon we focus our analysis only on 4 stations during the period from 01-Jan-2005 to 01-Jan-2022, covering 17 year of data. The selected stations are located in the south-west region of the lagoon and close to the southernmost inlet (Chioggia Cittá-Vigo), in front of the central inlet (Malamocco Porto), close to the Grand Canal and behind the northernmost inlet (Punta Salute), and in the northernmost region of the lagoon (Diga Nord Saline). Furthermore, since we are interested in a statistical characterization and diagnostic of extreme events in the Venice lagoon, while not primarily focusing in associating the value of the two metrics to a specific cause, we used hourly data to find a good balance between time resolution and statistics in terms of extreme events occurred when the MoSE was operating or not, thus to have a reliable diagnostic and comparison. In a further study we will focus both on higher time resolution and additional parameters in order to directly investigate how the two metrics can be used for forecasting purposes.

## Data Availability

The tidal data used in this study are made available at https://www.comune.venezia.it/it/content/centro-previsioni-e-segnalazioni-maree from the Centro Previsione e Segnalazione Maree - Protezione Civile, Venice, Italy, under the License Creative Commons Attribution-NonCommercial-ShareAlike 3.0 Italy (CC BY-NC-SA 3.0 IT).
