# Author Correction: Dynamical diagnostic of extreme events in Venice lagoon and their mitigation with the MoSE

**DOI:** 10.1038/s41598-023-40476-z

**Published:** 2023-08-21

**Authors:** Tommaso Alberti, Marco Anzidei, Davide Faranda, Antonio Vecchio, Marco Favaro, Alvise Papa

**Affiliations:** 1 Istituto Nazionale di Geofisica e Vulcanologia, via di Vigna Murata 605, 00143 Rome, Italy; 2 Marine Archaeology Research, 30100 Venice, Italy; 3 Laboratoire des Sciences du Climat et de l’Environnement, CEA Saclay l’Orme des Merisiers, UMR 8212 CEA-CNRS-UVSQ, Université Paris-Saclay & IPSL, 91191 Gif-sur-Yvette, France; 4 London Mathematical Laboratory, 8 Margravine Gardens, London, W6 8RH UK; 5 LMD/IPSL, Ecole Normale Superieure, PSL Research University, 75005 Paris, France; 6 Radboud Radio Lab, Department of Astrophysics/IMAPP-Radboud University, Nijmegen, The Netherlands; 7 LESIA, Observatoire de Paris, Universite PSL, CNRS, Sorbonne Universite, Universite de Paris, 5 Place Jules Janssen, 92195 Meudon, France; 8 Centro Previsioni e Segnalazioni Maree, 30100 Venice, Italy

Correction to: *Scientific Reports* 10.1038/s41598-023-36816-8, published online 28 June 2023

The Funding section in the original version of this Article was omitted. The Funding section now reads:

“This research was funded by Pianeta Dinamico Project, directed by INGV under the umbrella of the Italian Ministry of Research and SAVEMEDCOASTS2 Project (number 874398 www.savemedcoasts2.eu) funded by the European Commission through the DG-ECHO.”

The original Article has been corrected.

